# Machine Learning Based on Diffusion Kurtosis Imaging Histogram Parameters for Glioma Grading

**DOI:** 10.3390/jcm11092310

**Published:** 2022-04-21

**Authors:** Liang Jiang, Leilei Zhou, Zhongping Ai, Chaoyong Xiao, Wen Liu, Wen Geng, Huiyou Chen, Zhenyu Xiong, Xindao Yin, Yu-Chen Chen

**Affiliations:** 1 Department of Radiology, Nanjing First Hospital, Nanjing Medical University, Nanjing 210029, China; jiangliang0402@163.com (L.J.); troianll@163.com (L.Z.); 18110808328@163.com (Z.A.); 18851721636@163.com (W.G.); chenchenxiaoyou@163.com (H.C.); 2 Department of Radiology, Nanjing Brain Hospital Affiliated to Nanjing Medical University, Nanjing 210029, China; 13851424310@163.com (C.X.); nkmri@163.com (W.L.); 3 Department of Radiation Oncology, Cancer Institute of New Jersey, Rutgers University, New Brunswick, NJ 08901, USA; xiongzhenyu422@gmail.com

**Keywords:** diffusion kurtosis imaging, histogram analysis, machine learning, pathological grade

## Abstract

Glioma grading plays an important role in surgical resection. We investigated the ability of different feature reduction methods in support vector machine (SVM)-based diffusion kurtosis imaging (DKI) histogram parameters to distinguish glioma grades. A total of 161 glioma patients who underwent magnetic resonance imaging (MRI) from January 2017 to January 2020 were included retrospectively. The patients were divided into low-grade (*n* = 61) and high-grade (*n* = 100) groups. Parametric DKI maps were derived, and 45 features from the DKI maps were extracted semi-automatically for analysis. Three feature selection methods [principal component analysis (PCA), recursive feature elimination (RFE) and least absolute shrinkage and selection operator (LASSO)] were used to establish the glioma grading model with an SVM classifier. To evaluate the performance of SVM models, the receiver operating characteristic (ROC) curves of SVM models for distinguishing glioma grades were compared with those of conventional statistical methods. The conventional ROC analysis showed that mean diffusivity (MD) variance, MD skewness and mean kurtosis (MK) C50 could effectively distinguish glioma grades, particularly MD variance. The highest classification distinguishing AUC was found using LASSO at 0.904 ± 0.069. In comparison, classification AUC by PCA was 0.866 ± 0.061, and 0.899 ± 0.079 by RFE. The SVM-PCA model with the lowest AUC among the SVM models was significantly better than the conventional ROC analysis (z = 1.947, *p* = 0.013). These findings demonstrate the superiority of DKI histogram parameters by LASSO analysis and SVM for distinguishing glioma grades.

## 1. Introduction

Cerebral gliomas are still the most common primary brain tumors. According to the World Health Organization (WHO), cerebral gliomas are classified into four grades based on pathological appearance [1]. Preoperative glioma grading has important reference value for therapeutic decisions and prognosis [2,3]. The histopathology of resection specimens after surgical intervention, as the gold standard for the classification of glioma, is still recognized as the most accurate approach to determine the aggressiveness of glioma. However, robust and reliable noninvasive tumor grading is essential for follow-up of suspected low-grade glioma (LGG), for evaluation of surgical methods, for optimal care of patients who are not eligible for surgery, and for those being monitored for potential tumor recurrence.

The magnetic resonance imaging (MRI) technique has been widely used in the non-invasive diagnosis, management and follow-up of gliomas in clinical practice [4,5,6]. Conventional MRI techniques, including T1-weighted (T1), post-contrast enhanced T1-weighted (T1-Gd), T2-weighted, and T2-FLAIR images, could provide anatomical characteristics of brain tumors [7]. With the advancement of MRI imaging, diffusion-weighted imaging (DWI) [8,9,10] and diffusion tensor imaging (DTI) [11,12], which could provide further tumoral pathophysiology information, have been widely used in glioma grading.

DTI can reflect the anisotropic diffusion features of water molecules in vivo [13]. However, diffusion kurtosis imaging (DKI), an extension of the DTI, can provide more precise information on tissue characteristics by quantifying the degree of deviation from the Gaussian diffusion behavior of the diffusion-induced signal decay [14,15]. The metrics that can be derived from DKI are the mean diffusivity (MD), fractional anisotropy (FA), mean kurtosis (MK), kurtosis fractional anisotropy (KFA), and mean kurtosis tensor (MKT). Different from a small region of tumor biopsy, the DKI-based analysis may provide more information for the whole 3D tumor area. Previous studies have demonstrated that DKI is a useful method for glioma grading [16,17]. Histogram analysis is a mathematical method that can more comprehensively estimate tumor biological characteristics, including intratumor heterogeneity. Previously, histogram analysis of DKI stratified glioma according to 2016 WHO glioma grades [18] and distinguished between LGG and high-grade glioma (HGG) [19]. Although DKI is effective in differentiating glioma grades [20,21], these studies mentioned above mainly analyzed the value of DKI parameters in glioma differentiation, respectively, and did not consider the interaction of multiple histogram parameters.

Recently, machine learning (ML) has been used for key feature training and recognition and for group classification [22,23]. Bisdas et al. demonstrated that the grading accuracy achieved by DKI biomarkers was 78.1% using the support vector machine (SVM) method [24]. However, when there is considerable noise and redundancy in the data, the classification accuracy and convergence speed of the SVM algorithm will decrease. In response to the above problems, we tried some mainstream methods (principal component analysis (PCA), recursive feature elimination (RFE), minimum absolute shrinkage and selection operator (LASSO)) to simplify the data by reducing the feature dimensions and reflecting the original information as much as possible. On this basis, we applied the reduced DKI histogram parameters to establish the ML model using the SVM classifier, which has been demonstrated to provide better classification accuracy and generalized performance. Hence, the present study aimed to investigate the ability of different feature reduction methods in SVM-based DKI histogram parameters to distinguish glioma grades, and to compare their performances with results derived from conventional statistical methods.

## 2. Materials and Methods

### 2.1. Patients

Glioma patients admitted to the Nanjing First Hospital and Nanjing Brain Hospital from January 2017 to January 2020 were enrolled retrospectively. All glioma patients underwent preoperative MRI, including conventional MRI (T1WI, T2WI, fluid-attenuated inversion recovery (FLAIR), DWI, and T1-Gd) and DKI. Patients with corticosteroid or antibiotic treatment and previous brain surgery were excluded. According to the 2016 WHO criteria [1], a total of 161 patients were divided into LGG and HGG. All patients in this study provided written informed consent before the examination. The study was approved by the local ethics committee of Nanjing Medical University.

### 2.2. MRI Protocols

Preoperative MRI was performed on a 3.0-tesla MRI scanner (Magnetom Verio, Siemens Medical Solutions, Erlangen, Germany). The MRI protocol included T1WI, T2WI, FLAIR, DWI and contrast-enhanced MRI. The DWI protocol was as follows: single-shot spin-echo echoplanar imaging sequence, TR, 4500 ms; TE, 100 ms; acquisition matrix, 128 × 128; FOV, 256 mm × 256 mm; FA, 90°; slices, 20; section thickness, 5 mm; b values, 0 and 1000 s/mm^2^. The DKI protocol was as follows: ep2d_diff sequence, TR, 3000 ms; TE, 109 ms; acquisition matrix, 128 × 128; FOV, 256 mm × 256 mm; FA, 90°; slices, 15; section thickness, 4 mm; b values, 0, 500, 1000, 1500, 2000 and 2500 s/mm^2^, 30 diffusion directions. The T2WI protocol was as follows: turbo spin-echo imaging sequence, TR 4000 ms, TE 93 ms, acquisition matrix, 320 × 224; FOV, 256 mm × 256 mm; FA, 90°; slices, 20; section thickness, 5 mm. The T1-Gd protocol was as follows: fast spin-echo imaging sequence, TR 250 ms, TE 2.48 ms, acquisition matrix, 256 × 256; FOV, 256 mm × 256 mm; FA, 90°; slices, 20; section thickness, 5 mm.

### 2.3. Data Processing and Delineation of Volumes of Interest (VOIs)

**Figure 1:**
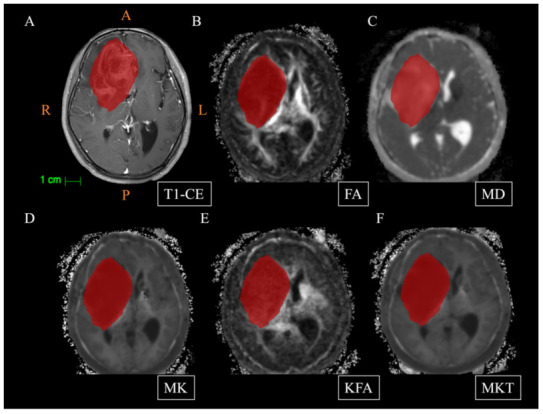
Delineation of volumes of interest (VOIs). A 60-year-old man with glioblastoma (WHO grade IV). (**A**): The lesion shows significant inhomogeneous enhancement on axial T1-weighted enhancement imaging. (**B**–**F**): fractional anisotropy (FA) parameter maps, mean diffusivity (MD) parameter maps, mean kurtosis (MK) parameter maps, kurtosis fractional anisotropy (KFA) parameter maps, and mean kurtosis tensor (MKT) parameter maps, respectively (the VOIs are outlined in red on each parameter image). A: Anterior; P: Posterior; R: Right; L: Left.

All diffusion images were evaluated before DKI parameter estimation to ensure that there was no apparent image distortion. The head motion and eddy current distortion correction was carried out using the FMRIB’s Diffusion Toolbox (FDT) (http://fsl.fmrib.ox.ac.uk/fsl/fslwiki/FSL, accessed on 11 August 2021). The Diffusion Kurtosis Estimator (DKE) (DKE 2.6, http://www.nitrc.org/projects/dke, accessed on 11 August 2021) was used for rigid-body coregistration for DKI images and spatial smoothing (a Gaussian smoothing kernel), to obtain parameter maps (FA, MD, MK, KFA, and MKT). For each case, VOIs were manually drawn on CE-T1WI maps using ImageJ software (version 1.51d, National Institutes of Health, Bethesda, MD, USA) by two neuroradiologists (C.X. and X.Y.) with more than 15 years of clinical experience, who were blinded to the patients’ histopathological results (Figure 1). Tumors including the solid portions, hemorrhage, cystic change, and necrosis were selected as VOIs and peritumoral edema was excluded. Then, based on the registration of DKI map histogram map with CE-T1WI map using the intensity-based automatic image registration method (one of the toolbox functions of MATLAB), the VOI on the CE-T1WI map was transformed on the DKI map histogram to obtain the VOI on the DKI map. Finally, these parameters can be derived from the DKI map: mean, minimum, maximum, variance, 25th percentiles (C25), 50th percentiles (C50), 75th percentiles (C75), skewness, and kurtosis. A total of 45 DKI features were obtained.

### 2.4. Feature Selection

There are certain interrelations between some variables, much noise and some degree of redundancy. Therefore, it is difficult to study the feature distribution in high-dimensional space. In this study, we used three different methods (PCA, RFE and LASSO) to select the discriminative features for glioma grading.

(1) PCA: PCA is a classic statistical method that can reduce the dimensionality of the original variable set by transforming to a new set of variables (the principal components) to summarize the features of the data [25]. Then, the first few principal components (PCs) are used in data analysis since they capture most of the variation in the original data set. In our study, the number of PCs was selected by taking the smallest number of PCs that accounted for at least 90% of the variance in the data.

(2) RFE: RFE is a SVM-based backward elimination method that iteratively eliminates the less important feature according to the weighting vectors of a SVM classifier and retrains the SVM until reaching a predefined number of features [26]. In recent years, many scholars have improved classification effectiveness in medical diagnosis by using this method [27,28]. The top-ranked features removed in the last iteration of SVM-RFE are the most important, while the bottom-ranked features are the least informative and are removed in the first iteration. We selected the top 20 percent ranked parameter features.

(3) LASSO: In LASSO regression, a single penalty parameter λ was applied equally to all regression coefficients to control the amount of regularization in the model. Better prediction accuracy may be achieved by allowing a differential amount of shrinkage. We used 10-fold cross-validation to tune a single penalty parameter. By constructing a penalty function and giving a small penalty to important features and a large penalty to unimportant features, LASSO could compress the coefficients of variables and make some regression coefficients become 0 to achieve the purpose of variable selection.

### 2.5. SVM Analysis

A SVM classification model based on three different feature selection methods to distinguish glioma grades from histogram parameters is presented in this paper. We used a Gaussian kernel function to map the initial input data into a high-dimensional space so that the two classes (LGG and HGG) of data become, as far as possible, linearly separable. Due to the limited number of patients, a nested leave-one-out cross validation (LOOCV) setting was used for model assessment. Moreover, due to the unbalanced data between the two groups, the LGG group was dynamically oversampled to reach a balance between the LGG and HGG groups during the training process.

### 2.6. Statistical Analysis

All continuous data are shown as the mean ± SD and were analyzed by using an independent-samples t-test or Fisher’s exact test, whereas categorical variables are presented as absolute and relative frequencies and were analyzed by using the chi-squared test to detect whereas variables differ between LGG and HGG group. *p* < 0.05 was considered significant. Logistic regression was used to identify the independent predictors of glioma grades among DKI parameters with statistical significance (*p* < 0.05). Receiver operating characteristic (ROC) curves were constructed to assess the area under the curve (AUC), and to determine the optimum threshold (Youden index) to differentiate LGG and HGG. Statistical analyses were performed using SPSS 26.0 software (SPSS Inc., Chicago, IL, USA). The performance prediction models based on SVM-PCA, SVM-RFE and SVM-LASSO were obtained using MATLAB (version R2013b) and evaluated by several metrics, including accuracy (ACC), sensitivity (SEN), specificity (SPC), AUC and F1 score. The differences in the performances from ROC analysis were evaluated according to Delong et al. [29].

## 3. Results

### 3.1. Comparisons of Clinical Data and DKI Histogram Parameters between LGG and HGG

A total of 161 patients with glioma, including LGG (*n* = 61) and HGG (*n* = 100) were enrolled. The clinical data and distribution of the histopathology are shown in Table 1. According to the 2016 WHO tumor classification, the LGG group included oligodendroglioma (*n* = 25) and diffuse astrocytoma (*n* = 36), and the HGG group included anaplastic astrocytoma (*n* = 25), anaplastic oligodendroglioma (*n* = 13), glioblastoma (*n* = 61) and gliosarcoma (*n* = 1). There were no significant differences in age and gender between the LGG and HGG groups. Comparisons of DKI histogram parameters between LGG and HGG are shown in Table 2.

### 3.2. Logistic Regression and ROC Analysis for Glioma Grading

**Figure 2:**
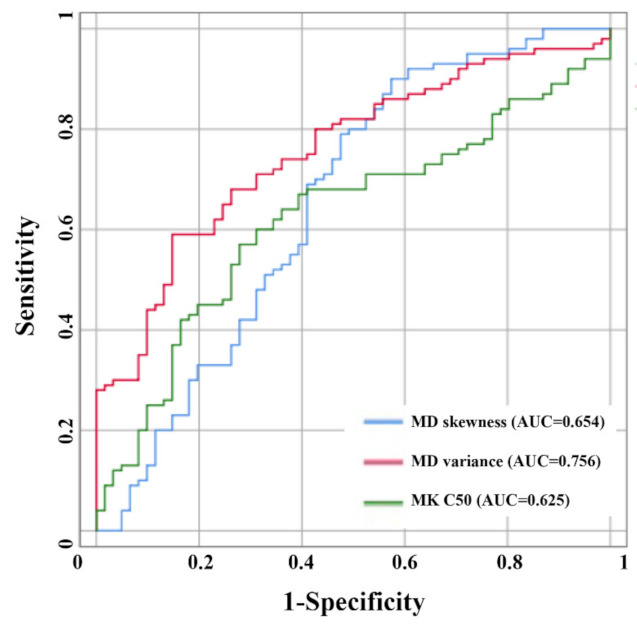
ROC curves of different DKI parameters for glioma grading. MD variance achieved the higher AUC (0.756), MD skewness and MK C50 showed lower values with AUCs of 0.654 and 0.625, respectively.

Logistic regression analysis demonstrated that FA skewness (*p* < 0.001), MD variance (*p* < 0.001), MD skewness (*p* = 0.047), MK C50 (*p* = 0.016) and KFA C25 (*p* < 0.001) were independent predictors for glioma grading (Table 3). The ROC analysis showed that MD variance, MD skewness and MK C50 could effectively distinguish LGG from HGG, particularly MD variance. The AUC, sensitivity and specificity of MD variance were 0.756, 0.590 and 0.852, respectively (Table 4, Figure 2).

### 3.3. SVM Analysis for Glioma Grading

**Figure 3:**
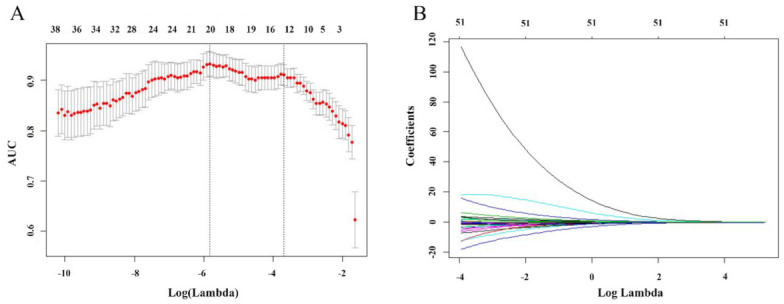
Feature selection using the least absolute shrinkage and selection operator (LASSO). (**A**) Tuning parameter (Lambda) selection in the LASSO model used 10-fold cross-validation via minimum criteria. (**B**) LASSO coefficient profiles of the 45 features. The optimal Lambda resulted in 12 nonzero coefficients.

**Figure 4:**
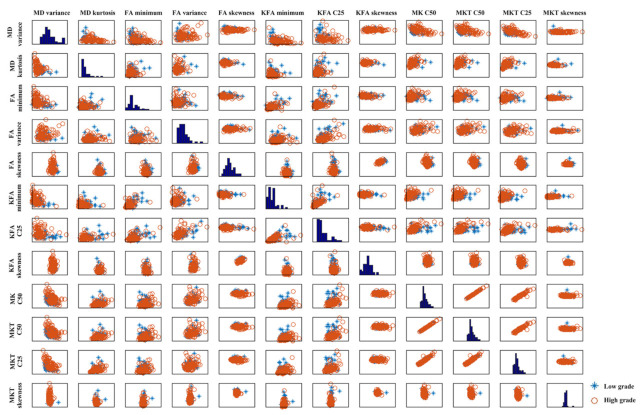
Scatter plots of the derived high-level features showing their pairwise correlations with respect to glioma grade. The figure is composed of scatter plots for each pairwise combination of the features (MD variance, MD kurtosis, FA minimum, FA variance, FA skewness, KFA minimum, KFA C25, KFA skewness, MK C50, MKT C50, MKT C25, MKT skewness). The sub axis of each of these scatter plots represent the range of the respective feature (either in x or y direction); the diagonal shows the histogram of the distribution of each feature in the data. Blue stars represent low-grade samples, whereas orange circles represent high-grade data.

**Figure 5:**
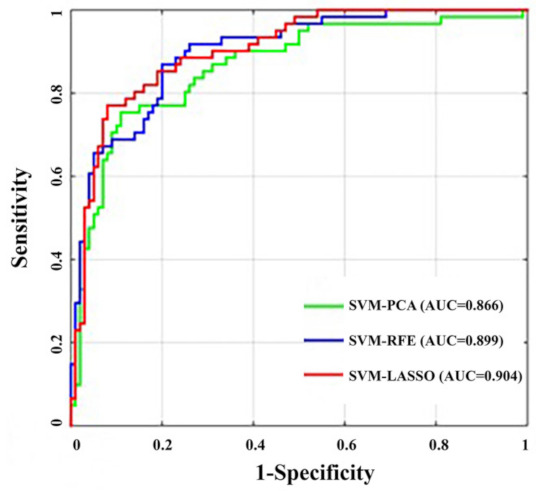
The ROC curves of different models for glioma grading. AUCs of AVM models for glioma grading was 0.866 by SVM-PCA, 0.899 by SVM-RFE and 0.904 by SVM-LASSO.

The SVM-PCA revealed that a maximum of ten principal components (PCs) explained over 90% of the variance in the data sets, and the results of the SVM-PCA model for glioma grading were 0.866 ± 0.061 AUC, 0.754 ± 0.148 sensitivity and 0.890 ± 0.123 specificity, respectively (Table 5). The SVM-RFE analysis revealed four SVM-RFE top-ranked features (MD maximum; MD variance; FA skewness; MK C50). The total performance of the SVM-RFE model for glioma grading was 0.899 ± 0.079 AUC, 0.869 ± 0.146 sensitivity and 0.800 ± 0.150 specificity, respectively (Table 5). The SVM-LASSO analysis (Figure 3) revealed ten optimal features (MD variance, MD kurtosis, FA minimum, FA variance, FA skewness, KFA minimum, KFA C25, KFA skewness, MK C50, MKT C25, MKT C50 and MKT skewness). Scatter plots of these high-level features are shown in Figure 4. There were significant correlations among these features as demonstrated by the correlation analysis, except for the weak correlation between KFA C25 (Figure 4). The SVM-LASSO model for glioma grading was 0.904 ± 0.069 AUC, 0.771 ± 0.152 sensitivity and 0.920 ± 0.094 specificity, respectively (Table 5). The ROC curve is shown in Figure 5. There were no differences in the AUC curves among SVM-PCA, SVM-RFE and SVM-LASSO (PCA vs. RFE: z = 1.473, *p* = 0.141; PCA vs. LASSO: z = 1.742, *p* = 0.082; RFE vs. LASSO: z = 0.346, *p* = 0.729). The SVM-PCA model with the lowest AUC was significantly better than the conventional ROC analysis (z = 1.947, *p* = 0.013).

## 4. Discussion

Preliminary studies have shown that DKI histogram parameters can be used as biomarker in glioma grading [19,30]. However, to our knowledge, using different feature selection methods to analyze DKI histogram parameters and differentiate LGG and HGG has not been studied. The results showed that SVM models using three methods (PCA, RFE and LASSO) had high accuracy in distinguishing glioma grades, and MD variance, FA skewness and MK C50 were found to be the optimal features for glioma grading. Importantly, the SVM methods had better performance than the conventional methods. Therefore, we firmly believe that DKI histogram parameters using SVM methods are valuable add-ons for differentiating glioma grades and can help to meet the exigent demand for noninvasive glioma grading before surgery.

Water diffusion in a homogeneous medium usually follows a Gaussian distribution. However, heterogeneous cellular and sub-cellular microstructures can substantially perturb the Gaussian distribution of diffusion displacement, leading to non-Gaussian diffusion [31]. Previous studies showed that the non-Gaussian MRI parameters (high b value, α and Dslow) were superior to Gaussian MRI parameters in glioma grading [10,32,33]. Sui et al. demonstrated that the non-Gaussian fractional order calculus diffusion model based on multi-b-value diffusion MRI imaging could differentiate low- and high-grade gliomas [34]. In addition, non-Gaussian diffusion model DKI could make diffusion MRI suitable for probing tissue microstructural complexity and heterogeneity. Specifically, MK values in patients with cerebral gliomas increased with higher glioma malignancy [35], which suggested that MK is able to help differentiate among glioma grades. Meanwhile, the values of MD and FA are not consistently helpful in glioma grading. Additionally, the sensitivity of DKI and DTI differs dramatically in largely isotropic tissues, such as gray matter (GM). Jensen et al. speculated that alterations in the structure of GM that may occur as a consequence of pathology would not change the fractional anisotropy but could shift the diffusional kurtosis [36,37]. These results indicated that DKI may be a potential candidate for glioma grading. Previous studies have indicated that DKI histogram parameters are helpful in glioma preoperative grading [19,21]. Their studies investigated the predictive value of a single DKI parameters based on conventional statistical methods. In our study, we sought to test this hypothesis beyond the conventional statistical approaches by using the SVM method. Our method enabled us to comprehensively evaluate histogram parameters as markers for glioma grading. SVM can obtain optimal results using available information and outperforms conventional methods in generalization ability for unseen data, including first-order statistics, shape/size-based features (2D, 3D), texture features, wavelet features, and histogram features [38]. Our findings demonstrated that the performance of the ML model using a SVM was better than conventional statistical methods for glioma grading. A SVM model with a nonlinear Gaussian kernel for all of the features was implemented for classification in our study. The SVM method leverages a powerful ML model that can produce nonlinear separation hyperplanes in the primal feature space and then avoids the assumption that the distribution of data is linearly separable [38].

To identify the best method for glioma grading, we carried out feature selection in three different manners (PCA, RFE, and LASSO). In the modeling process, the more input variables there are, the longer the modeling time will be. Moreover, the excess correlation variables could reduce the accuracy of the prediction. Therefore, PCA was utilized to compress the original variables. In our study, we retained more than 90% of the data variance in the eight-dimensional feature vector. PCA based on SVM can accelerate the modeling efficiency of the SVM algorithm. The recognition accuracy of the SVM-PCA model was 0.820. The advantage of RFE is that it can avoid redundancy between selected features while including features that provide complementary information for glioma grading [39]. The accuracy of the SVM-RFE model for glioma grading was similar to that of SVM-PCA (0.814 vs. 0.820). A study by Bisdas also used an ML method to predict glioma grades [24]. Their findings demonstrated that the grading accuracy achieved by DKI biomarkers was 87.1% for a method using texture analysis and SVM-RFE. The reason for this discrepancy may be the different compositions of the cohorts. In addition, we also used LASSO based on SVM to investigate the performance of glioma grading. LASSO is an outstanding method for feature selection, since it retains the desirable features of both subset selection and ridge regression [40]. It is suitable for analyzing sets of features in relatively small samples while avoiding overfitting [41]. This may be the reason why the LASSO method had slightly superior accuracy (0.857) in our study. It is worth noting that there were no differences in AUC among SVM-PCA, SVM-RFE and SVM-LASSO. In addition, we analyzed the performance of conventional statistical methods in glioma grading, and the results showed that MD variance, MD skewness and MK C50 could effectively distinguish between LGG and HGG, particularly MD variance. The SVM-PCA model with the lowest AUC among the ML modes was significantly better than the conventional ROC analysis. Our results indicated that ML methods can be useful in distinguishing glioma grades, and can outperform conventional statistical methods by a considerable margin.

It is worth pointing out that the RFE method identified four top-ranked features, and the LASSO method identified twelve optimal features. Interestingly, MD variance, FA skewness and MK C50 in DKI were found in both the RFE and LASSO methods. Especially, the MD variance and MK C50, which likely reflect the pathological characteristics of glioma, were also found by conventional statistical methods. The key factors of glioma pathological grade are mainly determined by the cell density and cell composition of the glioma [8,42]. In addition, we found that the MD variance and FA skewness values in LGG were significantly higher than those in HGG. These findings are partially consistent with those of previous studies using conventional statistical approaches. The results of MD in glioma grading are consistent with previous findings [42,43], while the FA values for glioma grading are still controversial. Some studies have found that the FA values of LGG and HGG are not significantly different, and cannot be used as an imaging marker in glioma grading [35,42,44]. Others have suggested that the FA values in LGG were significantly lower than those in HGG [19,45]. The results of our study were consistent with those of Rotkopf et al. [46]. The quantity and density of cells are higher in HGG than in LGG [17]. Specifically, glioblastomas often have obvious blood vessels and vascular tumor cells, resulting in increased FA values [16,47], and it still seems mechanistically plausible that HGG has a higher FA value than LGG. FA values may, therefore, differ depending on FA histogram parameters and the location of the region of interest (ROI). MK, the most representative parameter of DKI, represented the average value of kurtosis in all directions [48]. In our study, the MK C50 values of LGG were significantly lower than those of HGG, consistent with the previous findings [19,42,49]. Thus, the higher MK C50 values of HGG, and the increased kurtosis parameters might reflect a higher degree of tissue complexity of HGG17. Previous studies have shown that the increase in glioma grade, the complexity of glioma structure increases, and the MK value is closer to that of normal white matter [44]. In our study, although many DKI metrics between LGG and HGG were statistically significant, only MD variance, FA skewness and MK C50 were found to be the optimal biomarkers for glioma grading according to the ML method. Using the optimal features to distinguish the glioma grades reflects the effectiveness of the feature selection procedure and substantially simplifies its application, thereby maximizing its potential for clinical use.

The present study had some limitations. Since this study was conducted at a single center, the sample size was comparatively small and unbalanced, the number of LGG patients was relatively small, and its generalizability was limited. The results of our study require confirmation in a larger study. A larger sample size could also be used for deep learning to further verify the predictive value. Furthermore, the scan time and data post-processing time were relatively long. A newer and faster DKI post-processing method should be used to enable larger-scale implementation in clinical studies. Further ML is needed for automatic segmentation of glioma due to the long time required for ROI delineation.

## 5. Conclusions

In conclusion, our findings provide evidence for the diagnostic ability of SVM methods based on DKI histogram parameters in the prediction of glioma grading. We identified a small selection of biomarkers to distinguish LGG and HGG using SVM-RFE and SVM-LASSO. SVM-LASSO using DKI histogram parameters was feasible and performed even better than the conventional statistical method in distinguishing glioma grades. Therefore, we expect that DKI histogram parameters using SVM-LASSO have potential as new noninvasive biomarkers for glioma grading.

## Figures and Tables

**Table 1:** The clinical data and distribution of histopathological between low-grade glioma (LGG) and high-grade glioma (HGG).

Histopathological Characteristic	LGG (*n* = 61)	HGG (*n* = 100)	T/*χ^2^* Value	*p* Value
Age	48.77 ± 10.90	52.43 ± 13.98	4.608	0.006
Sex (male)	29 (47.54%)	58 (58.00%)	1.669	0.254
Histology	Oligodendroglioma (25, 40.98%) Diffuse astrocytoma (36, 59.01%)	Anaplastic astrocytoma (25, 25%) Anaplastic oligodendroglioma (13, 13%) Glioblastoma (61, 61%) Gliosarcoma (1, 1%)	-	

**Table 2:** The comparisons of DKI histogram parameters between low-grade glioma (LGG) and high-grade glioma (HGG).

	Mean	Minimum	Maximum	Variance	C25	C50	C75	Skewness	Kurtosis
FA									
LGG	0.06 ± 0.02 ^a^	0.01 ± 0.01 ^a^	0.23 ± 0.08 ^a^	0.03 ± 0.01 ^a^	0.04 ± 0.01 ^a^	0.06 ± 0.02 ^a^	0.08 ± 0.03 ^a^	1.23 ± 0.47	5.33 ± 2.17
HGG	0.07 ± 0.03 ^a^	0.02 ± 0.00	0.06 ± 0.02	0.03 ± 0.01	0.04 ± 0.02	0.06 ± 0.03	0.09 ± 0.03	0.81 ± 0.43	3.75 ± 1.32
T value	−0.959	2.734	0.640	−1.676	0.153	−1.199	−1.700	5.766	5.752
*p* value	0.339	0.007 *	0.523	0.096	0.879	0.232	0.091	<0.001 *	<0.001 *
MD									
LGG	2.50 ± 0.62	1.75 ± 0.53	3.72 ± 0.78	0.39 ± 0.18	2.24 ± 0.56	2.44 ± 0.62	2.75 ± 0.74	0.35 ± 0.79	3.83 ± 2.09
HGG	2.41 ± 0.58	1.73 ± 0.52	3.29 ± 0.66	0.25 ± 0.08	2.21 ± 0.55	2.40 ± 0.59	2.57 ± 0.62	0.65 ± 0.57	3.49 ± 1.64
T value	−0.948	0.174	−3.570	−6.564	0.301	−0.491	−1.638	−2.642	1.172
*p* value	0.344	0.862	<0.001 *	<0.001 *	0.764	0.624	0.103	0.010 *	0.243
MK									
LGG	0.53 ± 0.08	0.38 ± 0.06	0.84 ± 0.34	0.07 ± 0.03	0.48 ± 0.08	0.52 ± 0.08	0.57 ± 0.09	0.57 ± 0.76	4.07 ± 5.31
HGG	0.08 ± 0.05	0.37 ± 0.09	0.84 ± 0.29	0.08 ± 0.05	0.51 ± 0.10	0.57 ± 0.11	0.63 ± 0.12	0.11 ± 0.56	2.99 ± 1.43
T value	−2.962	0.968	0.000	−2.122	−2.783	−3.578	−3.691	4.392	1.926
*p* value	0.002 *	0.334	1.000	0.035 *	0.006 *	<0.001 *	<0.001 *	<0.001 *	0.056
KFA									
LGG	0.08 ± 0.04	0.02 ± 0.01	0.23 ± 0.13	0.03 ± 0.02	0.06 ± 0.03	0.08 ± 0.04	0.10 ± 0.05	0.95 ± 0.42	4.36 ± 1.47
HGG	0.07 ± 0.04	0.01 ± 0.01	0.22 ± 0.17	0.03 ± 0.02	0.05 ± 0.03	0.06 ± 0.04	0.09 ± 0.05	0.94 ± 0.45	4.26 ± 1.58
T value	1.508	3.244	0.378	−0.399	2.552	1.756	1.172	0.064	0.426
*p* value	0.134	0.001 *	0.706	0.691	0.012 *	0.081	0.243	0.949	0.670
MKT									
LGG	0.53 ± 0.08	0.38 ± 0.06	0.83 ± 0.34	0.07 ± 0.03	0.48 ± 0.07	0.52 ± 0.08	0.57 ± 0.86	0.56 ± 0.76	4.05 ± 5.28
HGG	0.57 ± 0.11	0.37 ± 0.09	0.83 ± 0.29	0.08 ± 0.05	0.51 ± 0.10	0.57 ± 0.11	0.63 ± 0.12	0.11 ± 0.56	2.98 ± 1.43
T value	−3.192	0.960	−0.020	−2.122	−2.785	−3.568	−3.689	4.360	1.916
*p* value	0.002 *	0.338	0.984	0.035 *	0.006 *	<0.001 *	<0.001 *	<0.001 *	0.057

Note: FA: fractional anisotropy; MD: mean diffusivity; MK: mean kurtosis; KFA: kurtosis fractional anisotropy; MKT: mean kurtosis tensor; ^a^ ×10^−3^ mm^2^/s; *: *p* < 0.05.

**Table 3:** The logistic regression analysis of *p* < 0.05 DKI histogram parameters.

	B	SE	Wald	OR (95%CI)	*p*
FA skewness	−2.669	0.678	15.509	0.069 (0.018–0.262)	<0.001
MD variance	16.087	3.259	24.360	96.95 (1.63–576.79)	<0.001
MD skewness	−0.0965	0.486	3.934	0.381 (0.147–0.989)	0.047
MK C50	−663.238	274.430	5.841	0.000 (0.000–0.000)	0.016
KFA C25	−56.660	14.118	16.107	0.000 (0.000–0.000)	<0.001

**Table 4:** ROC results of partial DKI histogram parameters.

	AUC	Cutoff	Sensitivity	Specificity
MD variance	0.756	0.31	0.590	0.852
MD skewness	0.654	0.49	0.600	0.689
MK C50	0.625	0.54	0.570	0.721

**Table 5:** The performance of different models for glioma grading.

	AUC	Accuracy	Sensitivity	Specificity	F1 Score
SVM-PCA	0.866 ± 0.061	0.820 ± 0.056	0.754 ± 0.148	0.890 ± 0.123	0.845 ± 0.122
SVM-RFE	0.899 ± 0.079	0.814 ± 0.055	0.869 ± 0.146	0.800 ± 0.150	0.908 ± 0.092
SVM-LASSO	0.904 ± 0.069	0.857 ± 0.081	0.771 ± 0.152	0.920 ± 0.094	0.863 ± 0.135

## Data Availability

The data presented in this study are available on request from the corresponding author. The data are not publicly available due to privacy considerations.
